# Saliency Changes Appearance

**DOI:** 10.1371/journal.pone.0028292

**Published:** 2011-12-05

**Authors:** Dirk Kerzel, Josef Schönhammer, Nicolas Burra, Sabine Born, David Souto

**Affiliations:** Section de Psychologie, Faculté de Psychologie et des Sciences de l'Éducation, Université de Genève, Geneva, Switzerland; Bielefeld University, Germany

## Abstract

Numerous studies have suggested that the deployment of attention is linked to saliency. In contrast, very little is known about how salient objects are perceived. To probe the perception of salient elements, observers compared two horizontally aligned stimuli in an array of eight elements. One of them was salient because of its orientation or direction of motion. We observed that the perceived luminance contrast or color saturation of the salient element increased: the salient stimulus looked even more salient. We explored the possibility that changes in appearance were caused by attention. We chose an event-related potential indexing attentional selection, the N2pc, to answer this question. The absence of an N2pc to the salient object provides preliminary evidence against involuntary attentional capture by the salient element. We suggest that signals from a master saliency map flow back into individual feature maps. These signals boost the perceived feature contrast of salient objects, even on perceptual dimensions different from the one that initially defined saliency.

## Introduction

Conspicuous objects in a visual scene attract attention in a bottom-up manner. For example, a lonely skier on an empty run is conspicuous because of the color, luminance, and motion contrast and will therefore rapidly attract attention. In the classical model of visual attention by Koch and Ullman [1], conspicuousness or saliency is represented for each location in the visual field in a two-dimensional, topographical map (see also [2], [3], [4], [5], [6]). The saliency map combines input from various feature maps (e.g., luminance, color, and motion maps) which encode the contrast between a stimulus and the surrounding context for the respective feature channels (e.g., on and off channels for the luminance intensity map). The spatial contrast in the feature maps is then fed into the unique saliency map. In the saliency map, information about the feature map in which a stimulus appeared salient is lost. The importance of the saliency map derives from its role in the bottom-up control of attention: the aforementioned models propose that attention scans locations in the visual field as indicated by the saliency map, starting at the most salient and moving toward less salient locations. Numerous studies have confirmed that attention may indeed be captured by salient stimuli [7], [8], [9]. In addition to bottom-up control of attention related to the saliency map, attention may be guided in a top-down manner by expectations, memory, or task requirements [10], [11], [12].

**Figure 1:**
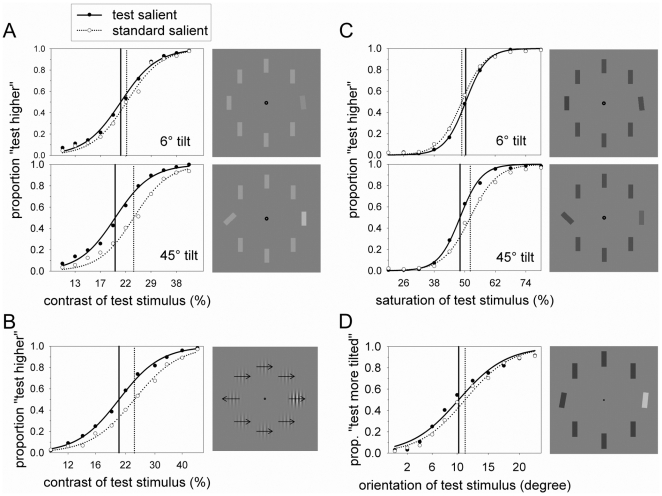
Experimental results and illustration of experimental trials (drawn to scale). Panels A, B, C, and D show the psychometric functions for Experiment 1 (Weber contrast), 2 (Michelson contrast), 3 (color saturation), and 5 (orientation of bar), respectively. The vertical lines represent the points of subjective equality (PSE) where the probability of judging the test to be brighter/more saturated/more tilted than the standard was 50%. Error bars have been omitted for clarity. Note that the illustrations on the right of each panel do not represent the PSE, but are random samples from the two conditions. The arrows in panel B illustrate the direction of motion, but were not shown in the actual experiments.

As outlined above, prominent saliency-based models have focused on the feedforward mechanisms by which saliency guides attention. In the present contribution, we are interested in effects of saliency on perception: How does saliency affect the perception of features at the salient location? While most computational models assume that saliency is calculated from individual feature maps, we ask whether saliency signals are fed back to the individual feature maps and enhance the local contrast of other, non-salient features. For instance, would the perceived contrast of a bar increase because it has an orientation different from the context that makes it salient? To investigate this question, we asked observers to judge which of two stimuli to the left and right of fixation had a higher contrast. One of the two stimuli had an orientation different from the context which made it salient (see Figure 1A). If the perceived contrast of the salient bar increased, a higher contrast of the non-salient, vertical bar would be necessary to match it. Conversely, a lower contrast of the salient element would be sufficient to match the contrast of the non-salient, vertical bar. To derive psychometric functions allowing for a test of this hypothesis, either the contrast of the salient or the non-salient bar varied between 11% and 43% while the contrast of the other bar was fixed at 22%.

## Experiment 1

### Methods

#### Participants

Twenty-two undergraduate students at the University of Geneva participated for course credit. All procedures were approved by the ethics committee of the University of Geneva (commission d'éthique de la Faculté de Psychologie et des Sciences de l'Éducation, Université de Genève) and written consent was obtained before the experiment started.

#### Apparatus

Stimuli were generated by a VISAGE system (Cambridge Research Systems, Rochester, UK) and presented at a refresh rate of 100 Hz on a 21″ CRT-monitor. The display had a resolution of 1024×768 (horizontal×vertical) pixels and was at a distance of 64 cm from the participant. Head movements were restrained by a chin rest.

#### Stimuli and Procedure

Participants fixated a black bull's eye with a diameter of 0.4°. Eight bars, 0.5° wide and 1.5° high, were presented on an imaginary circle at 4° from central fixation. The two bars on the horizontal axis passing through central fixation are referred to as test and standard stimulus and either one was tilted, while the remaining bars were upright. All bars had a Weber contrast of 22% with the exception of the test stimulus which had a contrast of 11%, 13%, 15%, 17%, 19%, 22%, 25%, 29%, 33,% 38, or 43%. Background luminance was 66 cd/m^2^. Each trial started with the presentation of the fixation mark for 500 ms. Then the target display was presented for 70 ms.

#### Design and Task

The 176 possible displays resulting from crossing the factors position of the standard stimulus (left, right), orientation of the tilted stimulus (left, right), tilted object (test, standard), level of tilt (6°, 45°), and contrast of test stimulus (11 contrast levels) were shown 4 times for a total of 704 trials. In the main experimental condition, 14 observers were told to indicate the stimulus on the horizontal axis that had a higher contrast by pressing a spatially corresponding key. In the inversed-judgment condition, 8 observers indicated the stimulus that had a lower contrast, but responses were re-coded to be consistent with judgments of higher contrast. Observers were instructed to ignore the orientation of the bars, to respond as accurately as possible, and to take their time to respond.

### Results and Discussion

Figure 1A shows the fit of a logistic function to the data from two main conditions: Trials in which the *test* stimulus was tilted and trials in which the *standard* stimulus was tilted. The test contrast with a 50% chance of being judged brighter than the standard was calculated separately for each tilted object and level of tilt. A two-way, within-subjects ANOVA (2 tilted objects ×2 levels of tilt) showed that when the test stimulus was tilted, the test contrast necessary to match the standard stimulus was lower than when the standard stimulus was tilted (20.3% vs. 23.1%), *F*(1,13) = 10.14, *p* = .007, suggesting that the tilted element was perceived to have a higher contrast. The effect was smaller with a tilt of 6° (20.8% vs. 22.2%) than with a tilt of 45° (19.7% vs. 23.9%), *F*(1,13) = 5.76, *p* = .032, showing that the increase in apparent contrast was larger with more salient stimuli, but separate t-tests showed that the effect of saliency was significant with the small, *t*(13) = 2.52, *p* = .026, and the large tilt, *t*(13) = 3.02, *p* = .01. Another ANOVA performed on the inversed-judgment condition replicated the effect of tilt (19.9% vs. 23.6%), *F*(1,7) = 25.60, *p* = .001, and the interaction of tilt and level of tilt (tilted by 6°: 21.2% vs. 22%, tilted by 45°: 18.7% vs. 25.2%), *F*(1,7) = 8.73, *p* = .021, ruling out simple response bias as an explanation (data not shown).

In the two following experiments, we wished to generalize the effect of saliency on appearance. In Experiment 2, we changed the perceptual dimension in which one stimulus appeared salient from orientation to direction of motion while observers continued to judge contrast. In Experiment 3, we probed the perception of color saturation with orientation singletons.

## Experiment 2

### Methods

Apparatus, stimuli, procedure, and design were the same as in Experiment 1 with the following exceptions. Eight Gabors with a spatial frequency of 1.5°/cycle and a space constant of 0.82° were presented at 6° from central fixation (center-to-center). The Gabors' carrier drifted at a velocity of 15°/s for 100 ms. The Michelson contrast of the test stimulus varied between 10%, 12%, 14%, 16%, 19%, 22%, 25%, 30%, 34%, 40%, and 47%, while the standard had a contrast of 22%. One Gabor drifted in a direction opposite to the remaining Gabors (the motion singleton). In pilot experiments, we measured the motion-induced position shift [13] and subsequently corrected the position of the singleton Gabor. Observers worked through 352 trials. Eleven undergraduate students participated.

### Results and Discussion

As shown in Figure 1B, the test contrast necessary to match the standard stimulus was lower when the test stimulus was the motion singleton than when the standard stimulus was the motion singleton (20.5% vs. 24.2%), *t*(10) = 4.03, *p* = .002. Thus, the perceived luminance contrast of both orientation and motion singletons is enhanced. In the following experiment we studied the perceived color saturation of orientation singletons.

## Experiment 3

### Methods

Apparatus, stimuli, procedure, and design were the same as in Experiment 1 with the following exceptions. The bars were red (CIE 1976: L = 29.6 cd/m^2^, u′ = 0.292, v′ = 0.482) with a saturation of 50% except for the test stimulus which had a variable saturation of 20%, 26%, 32%, 38%, 44%, 50%, 56%, 62%, 68%, 74%, or 80%. Zero percent saturation denotes dark gray bars (L = 29.6 cd/m^2^, u′ = 0.183, v′ = 0.448) and 100% denotes the exclusive output of the red gun (L = 29.6 cd/m^2^, u′ = 0.421, v′ = 0.522). The gradation was achieved by changing the contribution of the red gun to stimulus luminance. Observers were asked to judge saturation while ignoring orientation. Seventeen undergraduate students worked through 704 trials.

### Results and Discussion

Psychometric functions were fit to determine the test saturation necessary to match the standard stimulus of 50% saturation. A two-way ANOVA confirmed an interaction of tilt and level of tilt, *F*(1,16) = 23.34, *p*<.001. Unexpectedly, a higher saturation of the test object was necessary to match the standard when the test was tilted by 6° compared to when the standard was tilted by 6° and the test was upright (50.4% vs. 48.9%), *t*(16) = 2.23, *p* = .040, suggesting that the slight tilt decreased perceived saturation. We do not have a good explanation for the reversal, but speculate that the result is spurious. Consistent with the previous experiments, a lower saturation of the test object was sufficient to match the standard when the test was tilted by 45° compared to when the test was upright and the standard was tilted (48.1% vs. 52.2%), *t*(16) = 2.72, *p* = .015. Thus, we find that changes in appearance occur not only for luminance contrast, but also for color saturation.

## Experiment 4

An obvious explanation for changes in the appearance of salient objects is that attention was attracted to the salient stimulus and caused the apparent contrast to change. Using a similar psychophysical paradigm as in the present study, it has been demonstrated that peripheral cues preceding the target by 120 ms increase the perceived contrast and saturation of the target [14], [15]. In order to test how attention was deployed across the two response-relevant elements to the left and right of fixation, we measured event-related potentials (ERPs) in the contrast judgment task of Experiment 1. In particular, we were interested in the N2pc component which is widely used as an index of attentional selection (e.g., [16], [17]). The N2pc is greatest at posterior sites in the N2 latency range, about 200–300 ms after stimulus onset. It is defined as a larger negative deflection in electrodes contra-lateral to the target compared to ipsilateral electrodes.

### Methods

Apparatus, stimuli, procedure, and design were the same as in Experiment 1 with the following exceptions. The stimuli were presented on an LCD screen with a background luminance of 20 cd/m^2^ at a distance of 85 cm from the participant. There were only five levels of test contrast (11%, 17%, 22%, 29%, 43%) and the tilt of the orientation singleton was always 45°. For 800 trials, participants did the contrast judgment task in short blocks of 40 trials. Subsequently, participants were asked to judge the direction of tilt of the orientation singleton when all stimuli had equal contrast (160 trials). Twenty-one undergraduate students participated, but the data from five had to be discarded due to flat psychometric functions, the prominence of alpha waves, saccades, or muscular artifacts.

A Biosemi (Amsterdam, The Netherlands) ActiveTwo amplifier system with 64 active AG/AgCL electrodes including horizontal and vertical electro-oculograms was used. The two earlobes were taken as online and offline references. Data were filtered online with a 0.1 Hz high-pass filter and a 100 Hz low-pass filter. In the analysis of ERPs, choice errors in the orientation discrimination tasks and trials with response times smaller than 200 ms and larger than 2000 ms were removed, which amounted to 1% of all trials. Baseline correction (−100 ms to stimulus onset) was performed before artifact exclusion. We also excluded blinks (Fpz±60 µV), ocular movements (HEOG±40 µV) and muscular artifacts (all electrodes±100 µV) in epochs from −100 to 350 ms, which amounted to 13% of all trials.

### Results and Discussion

**Figure 2:**
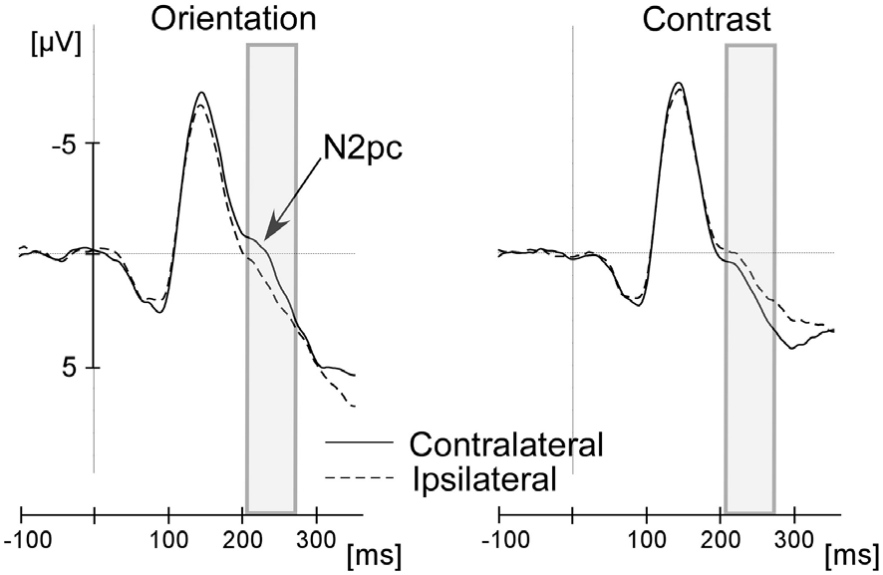
Results from Experiment 4. Grand average waveforms for electrodes PO7 and PO8 were averaged across positions ipsilateral and contralateral to the target. The panel on the left shows results from the control task in which observers judged the orientation of the tilted bar. The panel on the right shows results from the contrast judgment task. The N2pc component was clearly visible in the control task, but not in the contrast judgment task. The ERPs displayed in the figures were digitally low-pass filtered at 40 Hz.

We replicated the behavioral effect of saliency on contrast judgments (19.7% vs. 22.6%), *t*(15) = 3.68, *p* = .002 (data not shown). The orientation discrimination task was easy with only 3% choice errors. ERPs in the contrast judgment task were only analyzed when the contrast of test and standard was equal (160 trials), which was always the case in the orientation judgment task (also 160 trials). As shown in Figure 2, there was an N2pc in the 210–270 ms time range in the orientation judgment task (contralateral – ipsilateral = −1.09 µv), *t*(15) = 3.39, *p* = .004, whereas the opposite trend was visible in the contrast judgment task (0.59 µv), *t*(15) = 2.46, *p* = .027. The difference between tasks was significant, *t*(15) = 4.42, *p*<.001. Subsequently, a positive deflection occurred in the time interval from 250–300 ms for judgments of contrast (1.14 µv), *t*(15) = 3.33, *p* = .005. The late positive deflection has been related to the suppression of distractors (“distractor positivity”, [18], [19]). No distractor positivity appeared in the orientation discrimination task (−0.41 µv), *t*(15) = 1.34, *p* = .199. The difference between tasks was significant, *t*(15) = 4.18, *p*<.001.

The contrast judgment task does not provide evidence that attention was involuntarily drawn to salient objects because there was no N2pc to the orientation singleton. Instead, a significant deflection in the opposite direction occurred as part of a distractor positivity that peaked later, around 300 ms after stimulus onset. The distractor positivity suggests that observers attempted to suppress the salient singleton in the contrast judgment task. This finding may reflect our instruction to ignore orientation while judging contrast.

In a recent debate on the bottom-up or top-down control of attention, the absence of an N2pc component has been frequently associated with the lack of attentional capture. At the outset, Hickey, McDonald, and Theeuwes [20] reported an N2pc to salient color singletons when participants searched for a shape singleton, suggesting that salient stimuli involuntarily capture attention. In subsequent studies, this effect was not replicated in similar, but not identical paradigms [21], [22]. The absence of an N2pc, combined with a significant N2pc to the target stimulus, was taken to indicate that salient distractors do not attract attention in a bottom-up manner. This argument was also successfully applied when no target processing was necessary. In two studies [23], [24], an N2pc to salient, task-irrelevant stimuli in a cue display was observed. However, the N2pc to the cue display only occurred when the features of the cue matched the features of the subsequent target. Again, the absence of an N2pc to non-matching cue stimuli was taken to indicate that salient stimuli do not capture attention in a bottom-up manner. Rather, attentional capture (and the N2pc) depends on the match between the salient stimulus features and the search intentions of the observer.

While the absence of an N2pc is often interpreted to indicate the absence of attentional capture, we would nonetheless like to add a word of caution. Finding an N2pc implies that attention was captured, but the reverse is not always true. That is, not finding an N2pc does not necessarily mean that attention was not captured. For instance, it may be that attention was first allocated to the target and rapidly disengaged which may have prevented the N2pc to occur [25] (but see [26]). However, the absence of an N2pc and attentional capture with contrast judgments is very consistent with the task demands. There was no incentive for participants to pay more attention to the salient object because the task was to compare two objects on opposite sides of fixation. Therefore, attention had to be evenly spread across the two objects. In contrast, the attentional deployment to the orientation singleton required by the easy orientation discrimination task was sufficient to provoke an N2pc. We are therefore confident that we could have detected an N2pc if attention had been drawn toward the salient object in the contrast judgment task.

## Experiment 5

The final experiment was designed to rule out more complex forms of response biases. For instance, saliency on an irrelevant dimension may induce observers to also judge the object as more salient on a relevant dimension, without changes in appearance. If this was the case, saliency should also affect judgments when changes in appearance are implausible. In the present experiment, observers judged which of two bars was more tilted while one of them was a luminance singleton. It is implausible that saliency would change the perception of tilt because increasing the response or contrast gain of orientation-selective neurons does not result in increased tilt, but sharper tuning curves or higher firing rates (for review, see [27]).

### Methods

Apparatus, stimuli, procedure, and design were the same as in Experiment 1 with the following exceptions. To camouflage aliasing of tilted lines, the stimuli were enlarged to 1.25°×3.75° and presented at 10° from central fixation. The context elements were upright. The standard had an inclination of 10° while the test had a variable inclination of 0° (upright), 2°, 4°, 6°, 8°, 10°, 12.5°, 15°, 17.5°, 20°, or 22.5°. The inclination was always to the right. All stimuli were dark gray except for either the standard or the test stimulus which were light gray. The Weber contrast of dark and bright gray was 50%. Observers were asked which of the two stimuli on the horizontal axis was more tilted while ignoring luminance. Observers worked through 440 trials. Twenty-two undergraduate students participated.

### Results and Discussion

A psychometric function was fit to determine the orientation of the test stimulus necessary to match the standard which had a fixed tilt of 10° (see Figure 1D). When the test stimulus was a luminance singleton, the orientation necessary to match the standard was not significantly different from when the standard was a luminance singleton (10.3° vs. 11.3°), *t*(21) = 1.6, *p* = .124. Therefore, we conclude that even more complex forms of response bias cannot explain the shifts in psychometric functions induced by saliency in Experiments 1–4.

## General Discussion

Our results show that saliency changes the appearance of the salient object. The perceived contrast or saturation of salient singletons was enhanced. This finding has important implications for the architecture of computational models of saliency that focus on the feedforward summation of spatial feature contrast in a saliency map. Our results suggest that there is feedback from the saliency map to individual feature maps which enhances feature contrasts for the object even on perceptual dimensions different from the one that initially defined saliency. In other words, a strong feature contrast along one perceptual dimension affects perception of feature contrasts along other dimensions. As a result, salient objects look even more salient.

Computational models simulating the integration of signals from individual feature maps into a common saliency map were primarily developed to model bottom-up guidance of attention through a visual scene. Attention is first directed to the most salient item determined by a winner-take-all (WTA) competition and then moves toward less salient locations. The model of Itti and Koch [2] is essentially feedforward with the exception of the mechanism of inhibition of return, proposed to bias the competition away from the previously attended locations. In contrast, other authors have proposed that a WTA process is responsible for directing attention through feedback connections that go all the way down the visual hierarchy, inhibiting signals arising from losing locations and enhancing those at winning locations in the visual field [4]. To cover effects of saliency on perception, such feedback mechanisms are important [28]. Our results may suggest that feedback mechanisms in computational models have to go beyond inhibition. An activation mechanism may also be at play to explain how saliency on one dimension can enhance apparent feature contrast on another dimension.

Further, perceived contrast was modulated by saliency in Experiment 4 in the absence of an N2pc component in the ERP which would have indicated involuntary capture of attention by the salient orientation singleton. As already mentioned, the results have to be interpreted with caution and based on one single manipulation we cannot fully exclude any attentional involvement in our perceptual effects. We note, however, that the aforementioned models do not necessarily predict that attention is always directed towards the most salient stimulus. Top-down mechanisms are envisaged that guide attention according to the observer's goals or task demands. Our task requirements may have prevented the deployment of attention to the most salient location because observers had to compare two objects on opposite sides of fixation.

Our results are in line with studies on visual search showing that different visual features are not processed independently. In visual search for a feature singleton, faster search times are noted when the target is redundantly defined along two feature dimensions. For instance, it may be that the target is green among red, vertical distractors and additionally, it is tilted. RTs to redundant targets are faster than to simple targets. The redundancy gain has been assumed to arise from the convergence of signals onto a unique saliency map. With a saliency map that sums activity from lower levels, activity corresponding to the redundant target is always more likely to reach a critical threshold than in any of the feeding feature maps [29]. Independent processing was excluded by showing that reaction times to redundant signals are faster than predicted by the probability summation model or race model [29], [30], [31]. Further evidence for convergence was provided by studies measuring perceived saliency directly – i.e. by asking for saliency judgments. Targets which were salient in two feature maps (e.g., orientation and color) were perceived to be more salient than targets which were salient in just one feature map [7]. However, the perceived saliency of targets with two salient features was less than the sum of the perceived saliency of targets with only one of these features (see also [32]). It was hypothesized that the non-additivity was accounted for by cells that respond to more than one feature dimension [33] such that the activation from a feature combination does not simply add up. However, a recent study using an indirect measure of saliency, gaze location in a natural scene, concluded that the effects of saliency are linearly summed across luminance and color [34].

Further, we would like to discuss limitations of the present study which may have reduced the changes in appearance. First, our displays were relatively sparse. The saliency of our singletons may have been higher if a texture had been used [35]. However, we wanted to avoid effects of collinearity [36]. In our case, collinear facilitation may predict contrast enhancement for non-salient stimuli (i.e., test or standard) that are aligned with the context. However, the horizontal separation between test and standard and the closest context element was 1.2°, well beyond the limits of collinear facilitation (about 0.2° in [36]). Further, the saliency of the orientation or motion singleton may have suffered from the concomitant variation of contrast or saturation which produced an additional singleton. However, variation of contrast or saturation was necessary in order to measure perceived contrast. Thus, the setup of our experiments may have underestimated the size of changes in appearance.

Two different neural mechanisms may account for the spread of saliency from one perceptual dimension to the other. The first mechanism is feedback from higher-level areas to V1 (reviewed in [37]) that enhances the neural activity of salient elements. There are several areas in which activity correlates with stimulus saliency that could be considered as instantiations of a saliency map, such as the lateral intraparietal area, the frontal eye fields or a network of interacting areas implicated in eye movement target selection (reviewed in [38]). Most of these maps are thought to represent saliency independently of specific features. Therefore, signals may feed back to the individual feature maps in a way that is object-based or location-based. That is, they would not only target a specific feature map, but modulate all maps pertaining to a selected object or location. Alternatively, Li [5] proposed a model allowing the activity of V1 cells to provide a saliency map, without further need of combining separate feature saliency maps into a master saliency map. In this framework, the neural representation of context elements sharing a visual feature may be inhibited due to iso-feature suppression arising from long-range lateral interactions in V1 [5]. The iso-feature suppression spares the element different from the others, thereby making the stimulus salient. Further, we know from monkey physiology that at least V1, V2, and V3 neurons are often sensitive to more than one feature [39], [40], [41], [42]. If we suppose that conjunctive cells receive inhibitory input not only from similar conjunctive cells but also from single feature cells, they should receive less iso-feature suppression even if only one of the features is salient. Assuming further that conjunctive cells contribute to perception of both feature contrasts, less suppression of their overall response should enhance the perceived feature contrast on both features.

In conclusion, changes of appearance indicate that saliency could be the result of a recurrent calculation in which feedback from later stages influences early stages (i.e., the feature or channel stage). Further modeling and experimental efforts are needed to understand the time-course of this interaction, as well as the importance it might have for building an accurate model of saliency.

## References

[1] Koch C, Ullman S (1985). Shifts in selective visual attention: towards the underlying neural circuitry.. Hum Neurobiol.

[2] Itti L, Koch C (2001). Computational modelling of visual attention.. Nat Rev Neurosci.

[3] Wolfe JM (1994). Guided Search 2.0 A revised model of visual search.. Psychon Bull Rev.

[4] Tsotsos JK, Culhane SM, Kei Wai WY, Lai Y, Davis N (1995). Modeling visual attention via selective tuning.. Artif Intell.

[5] Li Z (2002). A saliency map in primary visual cortex.. Trends Cogn Sci.

[6] Treisman AM, Gelade G (1980). A feature-integration theory of attention.. Cognit Psychol.

[7] Nothdurft H (2000). Salience from feature contrast: additivity across dimensions.. Vision Res.

[8] Theeuwes J (1991). Cross-dimensional perceptual selectivity.. Percept Psychophys.

[9] Donk M, van Zoest W (2008). Effects of salience are short-lived.. Psychol Sci.

[10] Tatler BW, Hayhoe MM, Land MF, Ballard DH (2011). Eye guidance in natural vision: Reinterpreting salience.. J Vis.

[11] Folk CL, Remington RW, Johnston JC (1992). Involuntary covert orienting is contingent on attentional control settings.. J Exp Psychol Hum Percept Perform.

[12] Müller HJ, Reimann B, Krummenacher J (2003). Visual search for singleton feature targets across dimensions: Stimulus- and expectancy-driven effects in dimensional weighting.. J Exp Psychol Hum Percept Perform.

[13] De Valois RL, De Valois KK (1991). Vernier acuity with stationary moving Gabors.. Vision Res.

[14] Fuller S, Carrasco M (2006). Exogenous attention and color perception: performance and appearance of saturation and hue.. Vision Res.

[15] Carrasco M, Ling S, Read S (2004). Attention alters appearance.. Nat Neurosci.

[16] Eimer M (1996). The N2pc component as an indicator of attentional selectivity.. Electroencephalogr Clin Neurophysiol.

[17] Luck SJ, Hillyard SA (1994). Spatial filtering during visual search: Evidence from human electrophysiology.. J Exp Psychol Hum Percept Perform.

[18] Hickey C, Di Lollo V, McDonald JJ (2009). Electrophysiological indices of target and distractor processing in visual search.. J Cogn Neurosci.

[19] Sawaki R, Luck SJ (2010). Capture versus suppression of attention by salient singletons: electrophysiological evidence for an automatic attend-to-me signal.. Atten Percept Psychophys.

[20] Hickey C, McDonald JJ, Theeuwes J (2006). Electrophysiological evidence of the capture of visual attention.. J Cogn Neurosci.

[21] Wykowska A, Schubö A (2010). On the temporal relation of top-down and bottom-up mechanisms during guidance of attention.. J Cogn Neurosci.

[22] Töllner T, Müller HJ, Zehetleitner M (2011). Top-down dimensional weight set determines the capture of visual attention: Evidence from the PCN component.. Cereb Cortex.

[23] Eimer M, Kiss M (2008). Involuntary attentional capture is determined by task set: Evidence from event-related brain potentials.. J Cogn Neurosci.

[24] Lien MC, Ruthruff E, Goodin Z, Remington RW (2008). Contingent attentional capture by top-down control settings: converging evidence from event-related potentials.. J Exp Psychol Hum Percept Perform.

[25] Theeuwes J (2010). Top-down and bottom-up control of visual selection.. Acta Psychol.

[26] Eimer M, Kiss M (2010). The top-down control of visual selection and how it is linked to the N2pc component.. Acta Psychol.

[27] Reynolds JH, Chelazzi L (2004). Attentional modulation of visual processing.. Annu Rev Neurosci.

[28] Soltani A, Koch C (2010). Visual saliency computations: mechanisms, constraints, and the effect of feedback.. J Neurosci.

[29] Krummenacher J, Müller HJ, Heller D (2002). Visual search for dimensionally redundant pop-out targets: parallel-coactive processing of dimensions is location specific.. J Exp Psychol Hum Percept Perform.

[30] Koene AR, Zhaoping L (2007). Feature-specific interactions in salience from combined feature contrasts: evidence for a bottom-up saliency map in V1.. J Vis.

[31] Turatto M, Mazza V, Savazzi S, Marzi CA (2004). The role of the magnocellular and parvocellular systems in the redundant target effect.. Exp Brain Res.

[32] Poirier FJAM, Gosselin F, Arguin M (2008). Perceptive fields of saliency.. J Vis.

[33] Kastner S, Nothdurft HC, Pigarev IN (1999). Neuronal responses to orientation and motion contrast in cat striate cortex.. Vis Neurosci.

[34] Engmann S, Hart B, Sieren T, Onat S, König P (2009). Saliency on a natural scene background: Effects of color and luminance contrast add linearly.. Atten Percept Psychophys.

[35] Schade U, Meinecke C (2011). Texture segmentation: Do the processing units on the saliency map increase with eccentricity?. Vision Res.

[36] Kapadia MK, Ito M, Gilbert CD, Westheimer G (1995). Improvement in visual sensitivity by changes in local context: Parallel studies in human observers and in V1 of alert monkeys.. Neuron.

[37] Lamme VA, Super H, Spekreijse H (1998). Feedforward, horizontal, and feedback processing in the visual cortex.. Curr Opin Neurobiol.

[38] Fecteau JH, Munoz DP (2006). Salience, relevance, and firing: a priority map for target selection.. Trends Cogn Sci.

[39] Livingstone M, Hubel D (1984). Anatomy and physiology of a color system in the primate visual cortex.. J Neurosci.

[40] Ts'o DY, Gilbert CD (1988). The organization of chromatic and spatial interactions in the primate striate cortex.. J Neurosci.

[41] Gegenfurtner KR, Kiper DC, Levitt JB (1997). Functional properties of neurons in macaque area V3.. J Neurophysiol.

[42] Gegenfurtner KR, Kiper DC, Fenstemaker SB (1996). Processing of color, form, and motion in macaque area V2.. Vis Neurosci.

